# Preparation, Characterization, Antioxidant Activities, and Determination of Anti-Alzheimer Effects of PLGA-Based DDSs Containing Ferulic Acid

**DOI:** 10.1021/acsomega.3c07289

**Published:** 2024-02-29

**Authors:** Kübra Nur Arınmış, H. Tuba Kıyan, A. Alper Öztürk

**Affiliations:** † Faculty of Pharmacy, Department of Pharmaceutical Technology, Anadolu University, Eskişehir 26470, Türkiye; ‡ Graduate School of Health Sciences, Faculty of Pharmacy, Department of Pharmaceutical Technology, Anadolu University, Eskişehir 26470, Türkiye; § Faculty of Pharmacy, Department of Pharmacognosy, Anadolu University, Eskişehir 26470, Türkiye

## Abstract

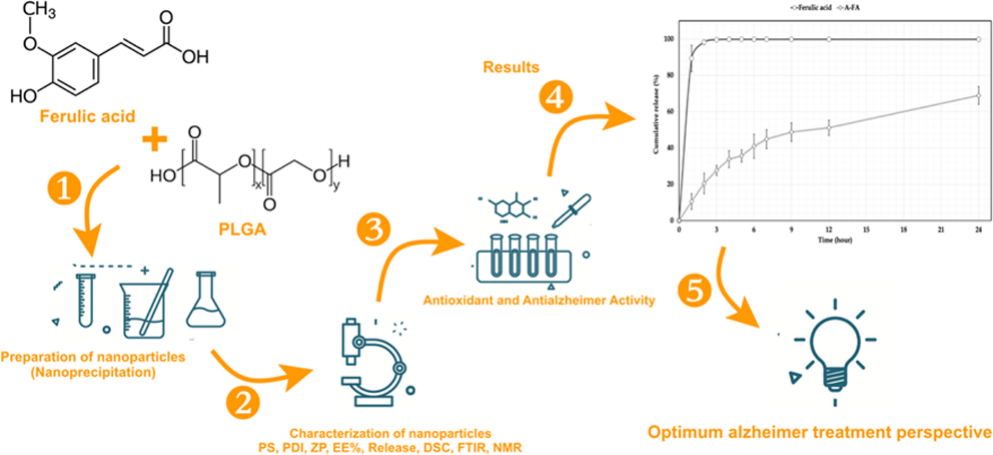

Nanoparticle (NP) systems have attracted the attention of researchers in recent years due to their advantages, such as modified release features, increased therapeutic efficacy, and reduced side effects. Ferulic acid (FA) has therapeutic effects such as anti-inflammatory, anti-Alzheimer’s, antioxidant, antimicrobial, anticancer, antihyperlipidemic, and antidiabetic. In this study, FA-loaded PLGA-based NPs were prepared by a nanoprecipitation method and the effect of varying concentrations of Poloxamer 188 and Span 60 on NP properties was investigated. FA-loaded A-FA coded formulation was chosen as optimum. High encapsulation efficiency has been achieved due to the low affinity of FA to the water phase and, therefore, its lipophilic nature, which tends to migrate to the organic phase. It was determined that the release of FA from the A-FA was slower than pure FA and prolonged release in 24 h. Antioxidant and anti-Alzheimer’s effects of A-FA coded NP formulation were investigated by biological activity studies. A-FA coded NP formulation showed strong DPPH free radical scavenging, ABTS cation decolorizing, and reducing antioxidant activity. Since it has both AChE inhibitor and antioxidant properties according to the results of its anti-Alzheimer activity, it was concluded that the formulation prepared in this study shows promise in the treatment of both oxidative stress-related diseases and Alzheimer’s.

## Introduction

The creation of novel pharmaceuticals has traditionally been centered on natural ingredients and their derivatives. Numerous studies have shown that oxidative stress is intimately related to the pathophysiological processes of ischemia-reperfusion injury, diabetes, kidney, liver, and colon diseases.^1^ Ferulic acid (FA) is a byproduct of 4-hydroxycinnamic acid, which can be discovered in a variety of foods, fruits, and beverages. It possesses antibacterial, anti-inflammatory, and antioxidant qualities that have been demonstrated by science. However, its limited clinical use, such as for the treatment of neurodegenerative diseases like Alzheimer’s disease (AD), is due to its poor ability to cross biological barriers (such as the blood–brain barrier, or BBB), its low bioavailability, and its quick elimination from the gastrointestinal tract after oral administration. As a result, novel nanotechnological methods are created to control the transport of FA within cells.^2^ A formulation development strategy based on nanotechnology is typically used to address the biopharmaceutical shortcomings of hydrophobic medications like FA.^3^

Advantages of using nanotechnology-based carriers to encapsulate drug active ingredients include better intracellular penetration, higher absorption–bioavailability, and regulated drug delivery.^4,5^ The creation of a novel pharmacological molecule is not only time-consuming but also costly and frequently unsuccessful. However, a more efficient strategy to enhance therapy may be to increase the bioavailability, targeting, efficacy, and safety of medications presently used in clinics. Researchers frequently develop drug delivery systems using nanocarriers for this reason.^6^ Regarding the usage of multifunctional nanopharmaceuticals, there are numerous instances in the literature and clinical settings, including those involving liposomes, polymeric nanoparticles (NPs), solid lipid NPs, quantum dots, iron oxide NPs, gold NPs, dendrimers, micelles, and carbon nanotubes.^7^ Today, NP-based medicines have a wide range of applications, and advances in nanotechnology offer new ways to approach treating medical conditions.^8^ The US Food and Drug Administration (FDA) and the European Medicines Agency (EMA) have both given their approval for the use of the well-known polymer poly(lactic-*co*-glycolic acid) (PLGA) in targeted chemotherapy administration in nanomedicine. In its role as a frequently used bulk and core material for NPs, PLGA exhibits a variety of advantageous and tuneable characteristics, including biodegradability, biocompatibility, and high tunability at the NP surface.^9^

When the literature is examined, it is seen that there are studies on FA-loaded drug carrier systems. Especially when FA-loaded polymeric systems in the literature are examined, the aims/biological effects of the studies are as follows: antitumor,^10,11^ cytoprotective,^12^ spinal cord injury,^13^ antimicrobial/antibacterial,^14,15^ antiproliferative and antiplatelet,^16^ wound healing and anti-inflammatory,^17,18^ tuberculosis,^19^ cytotoxic,^20^ diabetic wound healing,^21^ diabetes mellitus,^22^ antioxidant,^23^ bronchial asthma, and anti-inflammatory.^24^ When all of these polymeric systems are examined, it is seen that a relationship between oxidative stress and AD, which is the purpose of this study, has not been established and analyzed.

The most common type of senile dementia that affects older adults is called AD. It is characterized by extracellular β-amyloid (Aβ) containing neuritic plaques, tau-positive intracellular neurofibrillary tangles, and neuronal loss in certain brain regions, such as the neocortex, hippocampus, and basal forebrain, while the striatum and cerebellum are largely spared.^25^

The detrimental multidimensional phenomena known as oxidative stress (OS) is frequently referred to as the ″chemical silent killer″ since it lacks overt signs. There is currently no test that can be used to identify it. As a result, its harmful consequences can develop without giving the affected individual any guidance. Currently, OS is a significant issue since it is connected to the initiation and progression of hundreds of illnesses. Chemical defense by antioxidant molecules is one of the most effective and well-researched methods for reducing OS dangers to human health. Antioxidants act as sacrificed substances, preventing oxidants from damaging biomolecules. The body naturally produces antioxidants, which can also be obtained by eating certain foods and taking dietary supplements.^26^ Additionally obvious, oxidative stress or oxidative damage is frequently disregarded or seen as a result of the worsening of dementia symptoms. The amyloid-peptide’s activity, which has the potential to behave as both an antioxidant and a pro-oxidant molecule, is related to the regulation or beginning of oxidative stress. In addition, oxidative stress is linked to oxidative damage to proteins, nucleic acids, and lipids in susceptible cell populations, which ultimately results in neuronal death via many molecular processes. The development and testing of alternative therapeutic or preventive measures as possible or supplementary therapies for this debilitating neurodegenerative disease is enabled by the recognition of oxidative stress as a key component of AD.^27−29^

While the use of FA has been demonstrated to restore the activity of antioxidant enzymes, such as superoxide dismutase (SOD), catalase (CAT), and heme oxygenase-1 (HO-1), the antioxidant defense system is known to be damaged in AD. It has also been demonstrated that FA, both *in vitro* and *in vivo*, destabilizes the produced Aβ fibrils. Numerous studies have demonstrated how FA plays a crucial role in neuroprotection by regulating the expression of a number of important proteins, including p38, Hsp70, ERK1/2, foxo3a, and Akt. Furthermore, it has been observed that FA suppresses the inflammatory reactions in microglia triggered by lipopolysaccharides (LPS). Additionally, it has been demonstrated to alter β-secretase activity and enhance AD-like pathology in transgenic mouse model research. There have also been reports of FA restoring the potential of the mitochondrial membrane and inhibiting the activity of acetylcholinesterase (AChE).^30^

Although many studies have been conducted on FA in recent years, the number of studies examining and linking the antioxidant and anti-Alzheimer effects of the resulting formulations, polymeric systems, and drug delivery systems is quite limited.^31−33^ Additionally, when the literature was examined, it was seen that solid-state characterizations (analysis such as DSC, FT-IR, ^1^H NMR), which are very important for elucidating NP structures, were not performed.^34,35^ For all of these reasons, it was aimed in this study to prepare FA-loaded PLGA-based DDSs (Drug Delivery Systems) that can be used in the oral treatment of anti-Alzheimer and oxidative stress-related diseases in order to fill the gap in the literature.

## Experimental Section

### Materials

FA was purchased from Sigma-Aldrich (St. Louis, MO). PLGA [Resomer RG 503 H, Poly (*d*,*l*-lactide-*co*-glycolide), acid-terminated, lactide:glycolide 50:50, *M*_w_: 24.000–38.000], Span 60, ABTS, DPPH, BHT, ethanol, FeCl_3_, K_4_[Fe(CN)_6_]·3H_2_O, potassium phosphate dibasic, potassium phosphate monobasic, TCA, trehalose, and Vit C were purchased from Sigma-Aldrich (Germany). Poloxamer 188 is a kind gift from BASF (Germany). Acetone and Tween 80 were purchased from Merck (Germany). The remaining substances and reagents were all of the pharmaceutical and analytical grade.

### Preparation of Polymeric Nanoparticles

FA is a poorly soluble active ingredient.^36^ For this reason, as a result of literature research, PLGA NPs were prepared using the ’Nanoprecipitation’ method with minor modifications.^37−39^ In preliminary formulation studies, trial studies were carried out by changing the concentrations of surfactants named Span 60 and Poloxamer 188, and the optimum formulation was selected according to the appropriate results. In the relevant study, a polymer called Resomer RG 503 H, which is a special PLGA derivative, was studied. The contents of the prepared blank formulations and the formulation containing FA are shown in Table 1.

**Table 1:** Content of the Prepared Formulation^a^

	organic phase solution content				
code	PLGA	Span 60	FA	ACN	aqueous phase solution content
A-Blank	75 mg	25 mg	-	4 mL	10 mL, 0.5% (w/v) P-188 solution
B-Blank	75 mg	30 mg	-	4 mL	10 mL, 0.5% (w/v) P-188 solution
C-Blank	75 mg	35 mg	-	4 mL	10 mL, 0.5% (w/v) P-188 solution
D-Blank	75 mg	25 mg	-	4 mL	10 mL, 1.0% (w/v) P-188 solution
E-Blank	75 mg	30 mg	-	4 mL	10 mL, 1.0% (w/v) P-188 solution
F-Blank	75 mg	35 mg	-	4 mL	10 mL, 1.0% (w/v) P-188 solution
G-Blank	75 mg	25 mg	-	4 mL	10 mL, 1.5% (w/v) P-188 solution
H-Blank	75 mg	30 mg	-	4 mL	10 mL, 1.5% (w/v) P-188 solution
I-Blank	75 mg	35 mg	-	4 mL	10 mL, 1.5% (w/v) P-188 solution
A-FA	75 mg	25 mg	7.5 mg	4 mL	10 mL, 0.5% (w/v) P-188 solution

a PLGA: Resomer RG 503 H, FA: Ferulic acid, ACN: Acetone, and P-188: Poloksamer-188.

To prepare the formulations containing no active ingredient, 75 mg of exactly weighed Resomer RG 503 H and different amounts of Span 60 (25 mg, 30 mg, 35 mg) were dissolved in 4 mL of acetone selected as the organic phase. This obtained solution was dropped into different concentrations of 10 mL of Poloxamer 188 aqueous solution (0.5, 0.1, and 1.5% w/v) on a magnetic stirrer with a stirring speed of 100 rpm at a speed of 5 mL·h^–1^. After dropping, acetone was evaporated at room temperature in a magnetic stirrer (250 rpm), and centrifugation (11.000 rpm, 4 °C, 30 min) was applied to collect NPs from the resulting aqueous solution. After completing the centrifuge process to collect the NPs, the supernatant was removed and the collected NPs were dispersed in 15 mL of distilled water and centrifuged again. To thoroughly wash the NPs, this procedure was repeated three times.

To prepare the A-FA coded PLGA NP formulation containing FA, first 7.5 mg of FA, 75 mg of Resomer RG 503 H, and 25 mg of Span 60 were dissolved in 4 mL of acetone, which was selected as the organic phase. This obtained solution was dropped into Poloxamer 188 aqueous solution (0.5% w/v, 10 mL) on a magnetic stirrer with a stirring speed of 100 rpm at 5 mL.h^–1^ speed. After dropping, acetone was evaporated at room temperature in a magnetic stirrer (250 rpm), and centrifugation (11.000 rpm, 4 °C, 30 min) was applied to collect NPs from the resulting aqueous solution. After completing the centrifuge process to collect the NPs, the supernatant was removed and the collected NPs were dispersed in 15 mL of distilled water and centrifuged again. To thoroughly wash the NPs, this procedure was repeated three times.

### Particle Size, Polydispersity Index (PDI), and ζ-Potential

The Zetasizer Nano (Zetasizer Nano ZS, Malvern Instruments, Malvern, U.K.) was utilized to evaluate the particle size (PS) and polydispersity index (PDI) of nanoparticles using the dynamic light scattering technique. The PS and PDI of prepared NPs were determined by dispersing the formulation in distilled water. ζ-potential (ZP) was measured in a disposable folded capillary zeta cell at 25 °C room temperature and diluted with distilled water using the same instrument. For statistical analysis, each sample was measured three times, and the measurements’ average values and standard deviation were computed.^40,41^

### Determination of Cryoprotectant Effect and Storage Conditions

To determine the cryoprotectant effect of trehalose on the freezing process, 1 set of the optimum formulation coded A-FA was prepared. PS, PDI, and ZP of the freshly prepared formulation were measured. Then, centrifugation (11.000 rpm, 4 °C, 30 min) was applied to the prepared A-FA coded formulation. At this stage, trehalose solution (5.0%, w/v) was prepared and filtered through a 0.22 μm membrane filter. Following the washing processes of the centrifuged NPs, 2 mL of the trehalose solution prepared at 5.0% (w/v) concentration was added and vortexed. The formulation to which trehalose solution was added was divided into 8 equal parts in Eppendorf tubes (8 tubes), and trehalose solution at 5.0% w/v concentration was added to these tubes, respectively: 0 μL to the first tube, 100 μL to the second tube, 200 μL to the third tube, 300 μL to the fourth tube, 400 μL to the fifth tube, 600 μL to the sixth tube, 750 μL to the seventh tube, and 900 μL to the eighth tube. All tubes were frozen at −20 °C. All tubes were removed from the refrigerator after freezing, and after thawing, they were vortexed to disperse the NPs. PS, PDI, and ZP were measured for each tube. By comparing the results obtained throughout this study, the best trehalose ratio was determined to ensure that the NP properties did not change during the freezing process in the optimum formulation.^42−44^

To determine the cryoprotectant effect of trehalose on the lyophilization process and storage conditions, 1 set of optimally selected formulation, coded A-FA, was prepared. PS, PDI, and ZP of the freshly prepared formulation were measured. Then, centrifugation (11.000 rpm, 4 °C, 30 min) was applied to the prepared A-FA coded formulation. At this stage, trehalose solution (5.0%, w/v) was prepared and filtered through a 0.22 μm membrane filter. Following the washing processes of the centrifuged NPs, 5 mL of the trehalose solution prepared at 5.0% (w/v) concentration was added and vortexed. The formulation to which trehalose solution was added was divided into 12 equal parts in Eppendorf tubes (12 tubes), and trehalose solution at 5.0% (w/v) concentration was added to these tubes, respectively: 0 μL to the first tube, 100 μL to the second tube, 200 μL to the third tube, 300 μL to the fourth tube, 400 μL to the fifth tube, 600 μL to the sixth tube, 750 μL to the seventh tube, 900 μL to the eighth tube, 1050 μL to the ninth tube, 1300 μL to the 10th tube, 1450 μL to the 11th tube, and 1600 μL to the 12th tube. All tubes were frozen in the refrigerator at −20 °C and then lyophilized in a lyophilizer. As a result of lyophilization, 1 mL of distilled water was added to the lyophilized powder NP formulations and dispersed by vortexing. PS, PDI, and ZP were measured for each tube. By comparing the results obtained throughout this study, the best storage condition was determined due to the best trehalose ratio in storing the optimum formulation.^42−44^

### Gastrointestinal Stability Assessment

It is known that NPs made of hydrolytically degradable polymers such as PLGA degrade over time. pH and temperature have significant effects on long-term stability. A study was planned to examine the short-term stability of the A-FA coded PLGA NP formulation prepared within the scope of this study. Before starting this study, solutions that mimic gastrointestinal (GIS) fluids were prepared. These solutions are pH 1.2 hydrochloric acid (HCl) buffer (Solution 1), pH 6.8 phosphate buffer (Solution 2), and pH 7.4 phosphate buffer (Solution 3).

USP buffer solutions monograph was used when preparing these solutions. First, a 0.2 M potassium chloride (KCl) solution was prepared for the HCl buffer. First, 14.91 g of KCl was dissolved in water and diluted to 1000 mL with water. Fifty milliliters of this prepared KCl solution was taken and placed in a 200 mL measured bottle, 85 mL of 0.2 M HCl solution was added, and the required volume was completed with water. pH 1.2 HCl solution was controlled by pH measurement.

First, a 0.2 M monobasic potassium phosphate (KH_2_PO_4_) solution was prepared for the phosphate buffer. Then, 27.22 g of KH_2_PO_4_ was dissolved in water and diluted to 1000 mL with water. Fifty milliliters of this prepared KH_2_PO_4_ solution was taken and placed in a 200 mL measured bottle, 22.4 mL of 0.2 M NaOH solution was added, and the required volume was completed with water. pH 6.8 phosphate buffer solution was controlled by pH measurement. The only difference made when preparing pH 7.4 phosphate buffer is the amount of 0.2 M NaOH solution added, and this amount is 39.1 mL. Similarly, the pH 7.4 phosphate buffer solution was also controlled by pH measurement. The pH values of buffers were determined using a digital pH meter (Mettler Toledo S220 Seven Compact pH/lon Benchtop Meter).

These three solutions and distilled water (4 falcon tubes in total) were placed in a shaking water bath at a temperature of 37 ± 1 °C and a stirring speed of 50 rpm to mimic the gastrointestinal environment. One set of optimum formulation coded A-FA was prepared and completed to 4 mL after washing processes. One mL of the prepared NP dispersion was added to the medium incubated at 37 ± 1 °C, and samples were taken from each tube at the first hour, third hour, sixth hour, ninth hour, and 24th hour from the moment of addition, and PS measurements were made. In line with the measurement results, in which gastrointestinal environment PLGA NPs were more stable was determined.

### UV–Visible Spectrophotometric Method

Quantification of FA in *in vitro* studies was carried out using a UV spectrophotometer. For this purpose, two different validation studies were conducted for encapsulation efficiency (EE %) studies and *in vitro* release studies.^45−47^

#### UV–Visible Spectrophotometric Method for the Encapsulation Efficiency Test

The UV technique was used using a quartz cell and a UV-160A UV/vis recording spectrophotometer (Shimadzu) at 326 nm. In this study, analytical validation studies were conducted for FA. Accurately weighed 25 mg of FA were added to a 25 mL volumetric flask and dissolved in a 1:1 mixture of acetone and distilled water to create the standard solution, which had a final concentration of 1000 μg·mL^–1^. Using six concentrations of the standard solution (2–10 μg·mL^–1^), the calibration curve was produced. The linear regression analysis, which was computed using the least-squares regression approach, was used to assess the linearity. Repeatability (intraday) and intermediate precision (interday) were used to calculate the study’s precision. Assaying samples on the same day and at the same concentration allowed for the evaluation of repeatability. Comparing the assays conducted on three separate days allowed for the study of the intermediate precision. For every concentration, three sample solutions (4, 6, and 8 μg·mL^–1^) were made and examined. By recovering known quantities of FA reference standards that were added to the samples at the start of the procedure, the accuracy was ascertained. For this purpose, 10 mg of FA that had been precisely weighed was put into a 100 mL volumetric flask and dissolved in a 1:1 ratio of acetone to distilled water, resulting in a final concentration of 100 μg·mL^–1^. For the purpose of studying accuracy, solutions with concentrations of 4, 6, and 8 μg·mL^–1^ were produced from this solution. Each solution was created in triplicate and then examined. Regarding the method’s selectivity and specificity, the spectra obtained from the UV spectrophotometer were determined by looking at the overlap in the spectra of the samples obtained in the 200–800 nm range for FA and A-Blank (Blank formulation).

#### UV–Visible Spectrophotometric Method for the *In Vitro* Release Test

The UV technique was used using a quartz cell and a UV-160A UV/vis recording spectrophotometer (Shimadzu) at 307 nm. In this study, analytical validation studies were conducted for FA. Accurately weighed 25 mg of FA were added to a 25 mL volumetric flask and dissolved in PBS, pH 7.4, with 1% Tween 80 to create the standard solution, which had a final concentration of 1000 μg·mL^–1^. Using six concentrations of the standard solution (2–12 μg·mL^–1^), the calibration curve was produced. The linear regression analysis, which was computed using the least-squares regression approach, was used to assess the linearity. Repeatability (intraday) and intermediate precision (interday) were used to calculate the study’s precision. Assaying samples on the same day and at the same concentration allowed for the evaluation of repeatability. Comparing the assays conducted on three separate days allowed for the study of the intermediate precision. For every concentration, three sample solutions (4, 6, and 8 μg·mL^–1^) were made and examined. By recovering known quantities of FA reference standards that were added to the samples at the start of the procedure, the accuracy was ascertained. For this purpose, 10 mg of FA that had been precisely weighed was put into a 100 mL volumetric flask and dissolved in PBS, pH 7.4, with 1% Tween 80, resulting in a final concentration of 100 μg·mL^–1^. For the purpose of studying accuracy, solutions with concentrations of 4, 6, and 8 μg·mL^–1^ were produced from this solution. Each solution was created in triplicate and then examined. Regarding the method’s selectivity and specificity, the spectra obtained from the UV spectrophotometer were determined by looking at the overlap in the spectra of the samples obtained in the 200–800 nm range for FA and A-Blank (Blank formulation).

### Encapsulation Efficiency (EE, %)

The FA content of A-FA-loaded PLGA-based NP formulation was evaluated by directly extracting the drug from the NP formulation. The lyophilized NP was weighed with a precision balance to be 5 mg. Then, 1 mL of acetone:water (1:1, v/v) was added and vortexed for 5 min to dissolve the NPs. After the vortexing process, the prepared solution was filtered with a 0.22 μm membrane filter, and after the necessary dilutions, it was analyzed in a UV spectrophotometer at 326 nm for FA quantity determination. Encapsulation efficiency was expressed as (%EE) and calculated with eq 1.^48^
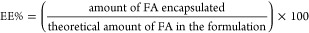
1

### *In Vitro* Release

The dialysis bag diffusion technique combined with a magnetic stirrer (IKA Labortechnik RT 15 S000, Germany) operating at a speed of 100 rpm was used to carry out the *in vitro* release experiments of the formulation. Briefly, 5 mg of FA and NP containing FA equivalent to 5 mg of FA was suspended in 2 mL of PBS, pH 7.4, containing 1% Tween 80 and transferred into a dialysis bag (dialysis tubing cellulose membrane with an average flat width of 33 mm (1.3 in.), *M*_w_ cutoff (MWCO): 14 000, D9652, Sigma-Aldrich). At 37 ± 1 °C, the dialysis bags were put into a beaker with 80 mL of dissolving medium. To prevent the release liquid from evaporating, the receptor compartment/beaker was sealed. Samples of the medium (3 mL) were withdrawn and replaced with fresh medium at 1, 2, 3, 4, 5, 6, 7, 9, 12, and 24 h. FA concentration in the samples was quantified by a UV spectrophotometer (307 nm). The *in vitro* release study was repeated three times for A-FA and pure FA, and then the results were calculated as mean ± SD. After that, the cumulative release of the results was plotted.

### *In Vitro* Release Kinetics

DDSolver software was employed to examine the kinetics of release. Data were submitted to the DDSolver application after getting the release profiles to identify the four crucial and well-liked criteria: coefficient of determination (Rsqr, *R*^2^, or COD), adjusted coefficient of determination (Rsqr_adj or *R*^2^_adjusted_), Akaike information criterion (AIC), and model selection criterion (MSC). The highest *R*^2^, *R*^2^_adjusted_, and MSC values and the lowest AIC values were used for evaluating different models.^49,50^

### Solid-State Characterization

#### Thermal Analysis

Differential scanning calorimetry (DSC, DSC-60, Shimadzu Scientific Instruments, Columbia, MI) was used to determine the thermal characteristics. Approximately 5 mg of the sample was weighed in an aluminum crucible and analyzed at a temperature range of 30 to 300 °C under an air flow of 50 mL·min^–1^ and a heating rate of 10 °C·min^–1^. In addition to the analysis of the A-FA, pure FA, A-Blank, and the physical mixture were analyzed for reference and comparison.^51^

#### Fourier-Transform Infrared Spectroscopy (FT-IR) Analysis

The Shimadzu IR Prestige-21 (Shimadzu Corporation, Kyoto, Japan) was used to record FT-IR spectra in the 4000–500 cm^–1^ wavelength range. Resomer RG 503 H (PLGA), pure FA, physical mixture, and blank formulation were also analyzed and used as references.^52^

#### Nuclear Magnetic Resonance (^1^H NMR) Analysis

The Ultra Shield CPMAS NMR (Brucker, Rheinstetten, Germany) was used for ^1^H NMR studies. Formulations were dissolved in deuterated acetone to create the samples. In addition, blank formulation, physical mixture, pure FA, and pure PLGA were examined and utilized as references.^52^

### Biological Activity

#### *In Vitro* Antioxidant Assays

##### ABTS Radical Cation Decolorization Assay

As a result of the oxidation of ABTS with potassium persulfate, the ABTS radical cation is formed. This reagent, which should be prepared fresh before each study, was diluted with ethanol to have an absorbance of 0.700 (±0.02) at a wavelength of 734 nm and was used to determine antioxidant activity. For the relevant reaction to occur, 990 μL of reagent solution was added to the A-Blank, A-FA containing 10 μL of the FA, and the FA solution at the same concentration and left for 5 min, and their absorbance values at 734 nm were measured. In this study, ascorbic acid (Vit C) was used as a positive control and ethanol was used as a blank for comparison. After spectrophotometric measurement, the % removal of the ABTS radical cation was calculated according to eq 2. In eq 2, *A*_control_ indicates the absorbance value of the ABTS reagent solution. In the relevant equation, *A*_sample_ indicates the absorbance of the reagent solution containing the sample formulation.^53^
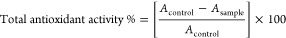
2

#### Reducing Power Activity

The improved method of determining the reducing power of samples was used. Samples were combined with 500 μL of phosphate buffer (200 mM, pH 6.6) and 500 μL of 1% potassium ferricyanide in varying concentrations. At 50 °C, the mixtures were incubated for 20 min. Then, 500 μL of 10% trichloroacetic acid was added to the mixtures after incubation, and the mixtures were then centrifuged at 4000 rpm for 10 min. The absorbance of the resulting solution was determined at 700 nm after mixing the upper layer (500 μL) with 200 μL of 0.1% ferric chloride and 500 μL of distilled water. Greater reducing power resulted from increased reaction absorbance. The term “IC_50_” refers to the concentration that produces an absorbance of 0.5 at 700 nm. Therefore, a lower IC_50_ suggested a higher reducing power.^53^

#### Free Radical Scavenging Assay (DPPH test)

The scavenging effects of the samples on DPPH free radicals were determined using a modified method. The free radical scavenging activities of the samples were expressed as a percentage of inhibition calculated according to eq 3. In eq 3, *A*_control_ is the absorbance of the control (containing all reagents except the test compound) and *A*_sample_ is the absorbance of the sample with added DPPH. The IC_50_ values were obtained by plotting the DPPH scavenging percentage of the sample against the sample concentration.^53^
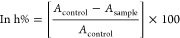
3

### Measurement of Anti-Alzheimer Effects by the *In Vitro* AChE and BuChE Inhibitor Activity Method

The Ellman test was used to determine the butyrylcholinesterase (BuChE) and acetylcholinesterase (AChE) enzyme inhibition activities of the test substances.^54^ Method modifications were made to ensure colorimetric clarity, and the modified method was applied. In this study, BuChE (isolated from horse serum, E.C.3.1.1.8.), AChE (isolated from type VIS *Electrophorus electricus* (electric eel) organism, E.C.3.1.1.7.), Ellman’s reagent, buffer solutions (potassium dihydrogen phosphate, potassium hydroxide), 5,5′-dithiobis (2-nitrobenzoic acid) (DTNB), gelatin, acetylcholine iodide (ATC), sodium hydrogen carbonate, butyrylcholine iodide (BTC), dimethyl sulfoxide (DMSO), and donepezil were used. Spectrophotometric measurements were carried out on a microplate reader (BioTek-Synergy H1 Microplate Reader) at a wavelength of 412 nm.

Experiments to determine the AChE and BuChE enzyme inhibition activities of the test substances were carried out in 96-well plates. Then, 2.5 μg·mL^–1^AChE and BuChE enzyme solutions, 0.075 M ATC and BTC solutions, 0.01 M DTNB solution, and 0.1 M phosphate buffer solution at pH 8, and test solutions at different concentrations were prepared for use in experiments.

These solutions were mixed to form two different test solutions. The first test solution was obtained by mixing 70 μL of phosphate buffer solution, 20 μL of DTNB solution, and 20 μL of enzyme solution (separate test solutions were prepared for each of AChE and BuChE) for each well. The other test solution was obtained by mixing 70 μL of phosphate buffer solution and 10 μL of ATC or BTC solution (a separate test solution was prepared for each of ATC and BTC) for each well. Test solutions were prepared in quantities sufficient for all 96 wells.

First, 20 μL of the test solution and 110 μL of the first test solution were added to the 96-well plate in 4 replicates for each concentration, mixed for 5 min, and incubated for 15 min at 25 °C. After the incubation, 80 μL of the second test solution was added to all wells, and a rapid mixing process was applied for 30 s. After the mixing process, the first spectrophotometric measurement was performed at a wavelength of 412 nm in a microplate reader (BioTek-Synergy H1 Microplate Reader). After waiting 5 min for the reaction to continue, the second spectrophotometric measurement was performed at the end of the period. By taking the difference in absorbance values between the two measurements, % inhibition rates were calculated with the help of eq 4. The explanations of the abbreviations used in eq 4 are as follows: *A*(*C*): Absorbance reading difference of the control well; *C*: Control (well only to which test solution is not added); *B*: Blank (well to which test solution and substrate are not added); *A*(*B*): Absorbance reading difference of the blank well; and *A*(*I*): Absorbance reading difference of the test solution.
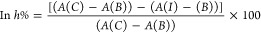
4

All of the data determined from the ‘*In vitro* antioxidant assays’ and ‘Measurement of the anti-Alzheimer effect by *in vitro* AChE and BuChE inhibitor activity method’ were analyzed using SigmaPlot software (Version 14.5), and the IC_50_ values were obtained.

## Results and Discussion

### Particle Size, Polydispersity Index (PDI), and ζ-Potential

PS, PDI, and ZP analysis results are presented in Table 2. Within the scope of this study, 9 blank formulations were first prepared to examine the effect of Poloxamer 188 concentration in the aqueous phase and Span 60 concentration in the organic phase on NP properties. When the literature was examined, the A-Blank coded formulation with a PS of 164.83 nm ± 1.68 was chosen as the optimum formulation without the active ingredient in order not to use too much excipient and to have the lowest particle size.^42^ PS, PDI, and ZP results of FA-loaded A-FA coded NP formulation were obtained as 174.70 nm ± 0.89, 0.113 ± 0.006, and −22.00 mV ± 0.56, respectively. According to the literature, it has been emphasized that the PS in active substance-encapsulated nanosystems may be larger than in blank formulations. This situation has been explained as the formation of a NP system by encapsulating the active drug substance of large molecule structures such as polymers, thus increasing the PS of the prepared NPs.^55^

**Table 2:** Particle Size, PDI, and ζ Potential Results

code	particle size (nm, mean ± SD)	PDI (mean ± SD)	ζ potential (mV, mean ± SD)
A-Blank	164.83 ± 1.68	0.093 ± 0.008	–18.87 ± 0.15
B-Blank	166.17 ± 1.08	0.089 ± 0.077	–25.27 ± 0.57
C-Blank	176.10 ± 1.68	0.111 ± 0.023	–25.63 ± 0.38
D-Blank	182.33 ± 2.64	0.072 ± 0.027	–23.55 ± 0.07
E-Blank	210.40 ± 6.56	0.081 ± 0.027	–26.77 ± 0.72
F-Blank	208.90 ± 3.05	0.144 ± 0.061	–27.23 ± 0.49
G-Blank	206.13 ± 1.44	0.034 ± 0.016	–28.80 ± 0.06
H-Blank	217.30 ± 2.95	0.131 ± 0.024	–25.67 ± 0.80
I-Blank	188.73 ± 1.08	0.117 ± 0.013	–27.20 ± 0.17
A-FA	174.70 ± 0.89	0.113 ± 0.006	–22.00 ± 0.56

In this study, both antioxidant activity and anti-Alzheimer activity were targeted. The ability of NPs to pass the blood–brain barrier is very important, especially in the treatment of AD. When the literature is examined, it is emphasized that a PS of less than 250 nm is an important feature for crossing the blood–brain barrier.^56^ In another study, it was emphasized that the PS was around 150 nm.^57^ In light of this information, it is thought that the A-FA coded NP system prepared in this study may be used in AD.

The homogeneity of the PS distribution in a certain nanosystem is shown by the PDI, which measures the quality of NP dispersion in the 0.0–1.0 range. When the PDI value is less than 0.1, it denotes great dispersion quality and shows that practically all of the NPs are around the same size. The PDI values of the prepared NPs must be less than 0.3 to be considered as the optimum value, but it has been reported in the literature that values less than 0.5 are also acceptable.^58^ As a result, it can be said that the formulation coded A-Blank and A-FA, prepared and chosen optimally in this study, is a monodisperse and high-quality system.

Negative ZP values were obtained in all NPs. The PLGA polymer in a neutral environment has a negative surface potential due to the terminal carboxyl groups in its structure, and this feature of PLGA explains the negative ZP values obtained in the prepared NPs.^59^ ZP values between −5.0 and −15.0 mV obtained in NP systems show that the NP system is in the limit region of flocculation, and values between −5.0 and −3.0 mV have been previously reported to be the maximum flocculation region for a NP system.^60^ In light of this information, it was concluded that the optimally selected A-FA coded formulation could be relatively stable, thanks to the ZP value obtained as −22.00 ± 0.56.

### Determination of Cryoprotectant Effect and Storage Conditions

The effect of different trehalose concentrations on PS, PDI, and ZP of A-FA in the freezing and lyophilization processes are presented in Tables 3 and 4, respectively. Freeze-drying has been recognized as a good technique to improve the long-term stability of colloidal NPs. The poor stability of these systems in aqueous media poses a real obstacle to the clinical use of NPs.^61^ When NPs are stored in aqueous media, they lose almost all of their advantages and encounter disadvantages such as physical instability (aggregation/particle fusion) and/or chemical instability (hydrolysis of polymer materials forming NPs, drug leakage from NPs, and chemical reactivity of the drug during storage).^62,63^ For this reason, water needs to be removed from the environment. In the lyophilization process, some special agents are added to prevent the NP formulation from being affected by drying stress (lyoprotectant) or freezing stress (cryoprotectant) and to increase its stability during storage. Sugar derivatives are the most often employed cryoprotectants for freeze-dried NPs in the literature. These agents include mannitol, trehalose, sucrose, and glucose.^61^

**Table 3:** Effect of Different Trehalose Concentrations on the Properties of A-FA Coded Formulation in the Freezing Process

condition	particle size (nm, mean ± SD)	PDI (mean ± SD)	ζ potential (mV, mean ± SD)
fresh formulation	173.70 ± 1.88	0.111 ± 0.022	–23.17 ± 0.58
Tube 1 (0 μL of trehalose solution)	265.10 ± 5.11	0.494 ± 0.041	–22.97 ± 1.40
Tube 2 (100 μL of trehalose solution)	189.77 ± 1.70	0.218 ± 0.015	–25.03 ± 0.57
Tube 3 (200 μL of trehalose solution)	186.30 ± 0.20	0.202 ± 0.034	–25.40 ± 0.62
Tube 4 (300 μL of trehalose solution)	176.20 ± 2.86	0.156 ± 0.012	–25.73 ± 1.17
Tube 5 (400 μL of trehalose solution)	174.77 ± 2.88	0.115 ± 0.004	–25.13 ± 0.23
Tube 6 (600 μL of trehalose solution)	174.08 ± 2.35	0.112 ± 0.049	–24.93 ± 1.29
Tube 7 (750 μL of trehalose solution)	172.77 ± 3.21	0.114 ± 0.002	–25.67 ± 1.64
Tube 8 (900 μL of trehalose solution)	174.93 ± 2.57	0.115 ± 0.047	–27.07 ± 1.37

**Table 4:** Effect of Different Trehalose Concentrations on the Properties of the A-FA Coded Formulation in the Lyophilization Process

condition	particle size (nm, mean ± SD)	PDI (mean ± SD)	ζ potential (mV, mean ± SD)
fresh formulation	173.70 ± 1.88	0.111 ± 0.022	–23.17 ± 0.58
Tube 1 (0 μL of trehalose solution)	532.50 ± 3.48	0.531 ± 0.033	–14.70 ± 0.70
Tube 2 (100 μL of trehalose solution)	361.47 ± 10.89	0.430 ± 0.044	–24.93 ± 0.42
Tube 3 (200 μL of trehalose solution)	350.47 ± 4.97	0.403 ± 0.037	–24.67 ± 1.04
Tube 4 (300 μL of trehalose solution)	288.43 ± 7.21	0.352 ± 0.019	–24.77 ± 1.20
Tube 5 (400 μL of trehalose solution)	267.50 ± 7.10	0.318 ± 0.076	–23.93 ± 0.32
Tube 6 (600 μL of trehalose solution)	239.47 ± 10.20	0.265 ± 0.029	–22.63 ± 0.78
Tube 7 (750 μL of trehalose solution)	238.60 ± 6.95	0.260 ± 0.030	–21.07 ± 0.25
Tube 8 (900 μL of trehalose solution)	252.03 ± 2.86	0.240 ± 0.027	–23.27 ± 0.71
Tube 9 (1050 μL of trehalose solution)	205.80 ± 0.97	0.201 ± 0.043	–23.40 ± 0.25
Tube 10 (1300 μL of trehalose solution)	187.62 ± 2.34	0.186 ± 0.035	–23.63 ± 0.44
Tube 11 (1450 μL of trehalose solution)	174.33 ± 1.27	0.169 ± 0.054	–24.13 ± 1.05
Tube 12 (1600 μL of trehalose solution)	174.06 ± 2.04	0.166 ± 0.091	–23.80 ± 0.45

After the analyses performed in this study, it was decided that 1450 μL of trehalose solution was sufficient to keep the particle size constant after the freeze-drying/lyophilization process. In the analysis in which 1450 μL of trehalose solution was added, the PS, PDI, and ZP values were like the values of the fresh formulation, and it was concluded that the use of 1450 μL of trehalose solution was sufficient to ensure that the properties of the fresh formulation did not change. Additionally, it was concluded that 400 μL of trehalose solution may be sufficient if only freezing is to be done.

### Gastrointestinal Stability Assessment

**Figure 1:**
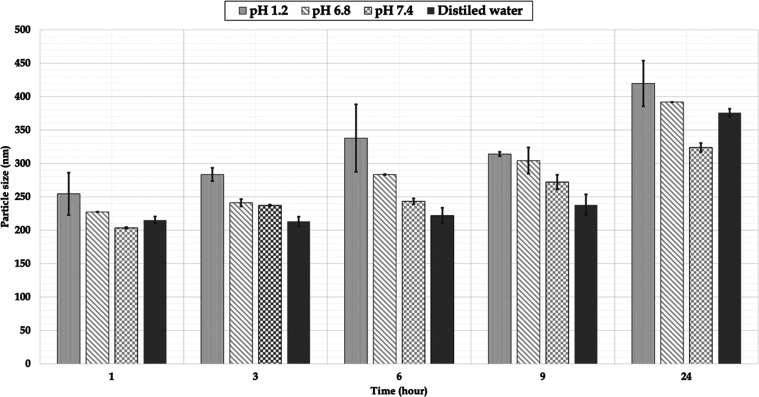
Gastrointestinal stability result graph of A-FA coded nanoparticle formulation.

The results of the study examining the short-term stability of the A-FA coded PLGA NP formulation in pH 1.2 HCl buffer, pH 6.8 phosphate buffer, pH 7.4 phosphate buffer, and distilled water, which mimics gastrointestinal fluids, for 24 h are presented in Figure 1. When the results of the analyses were examined, it was determined that the PSs of the NPs in pH 6.8, pH 7.4, and distilled water increased more slowly and to a lesser extent. On the other hand, the PS of the NPs in pH 1.2 HCl buffer increased more than the zero time, and a value of 419.7 nm ± 34.2 was obtained at the end of the 24th hour. It can be concluded that the A-FA coded NP formulation prepared in this study breaks down with rapid degradation in pH 1.2 HCl buffer and slow degradation in other environments.

### UV–Visible Spectrophotometric Method

After the devised UV–visible spectrophotometric technique was validated in acetone:distilled water (1:1, v/v), linearity of *y* = 0.0977*x* – 0.0018 (*r*^2^ = 0.9995) was found in a concentration range of 2–10 μg·mL^–1^. Relative standard deviation (RSD) values of less than 2% for repeatability and moderate accuracy led to the determination that the procedure was exact. The method’s recovery and accuracy were deemed adequate due to an RSD value of less than 2%. Accuracy values of 99.953% ± 0.512, 99.884% ± 0.171, and 99.935% ± 0.517 for concentrations of 4, 6, and 8 μg·mL μg·mL^–1^, respectively, were determined for the UV–visible spectrophotometric method. Blank formulations (A-blank) were photometrically analyzed between 200 and 800 nm in the selectivity research, and they did not produce an absorbance peak at 326 nm. LOD and LOQ were obtained as 0.226 and 0.685 μg·mL^–1^, respectively, and proved its suitability by being lower than the lowest concentration studied in linearity. As a result, routine and simultaneous FA determination can be accomplished using the simple and affordable approach suggested in this study.^64^

**Figure 2:**
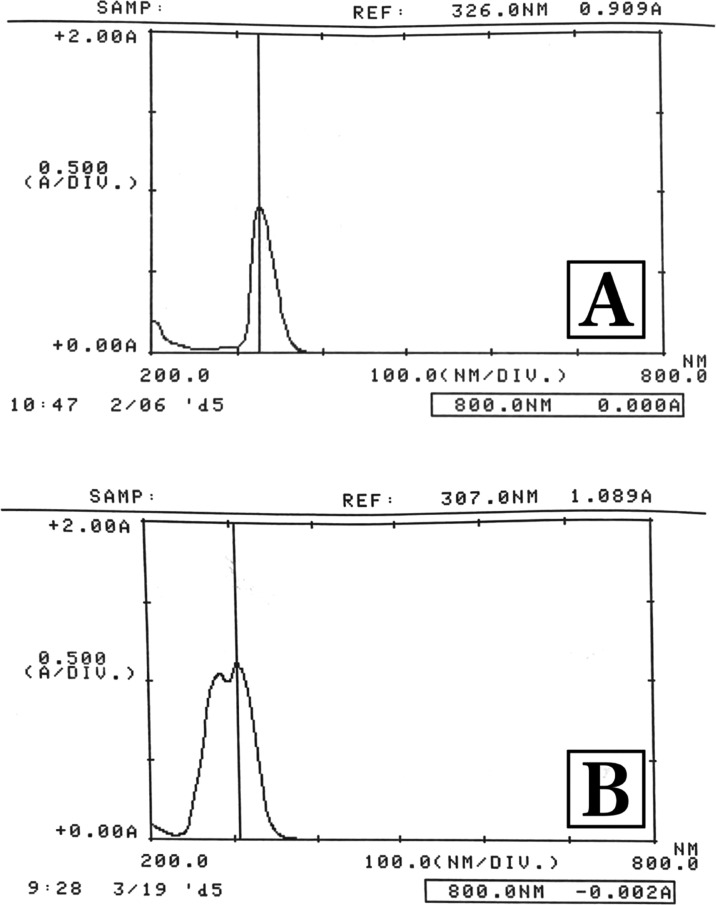
Spectra from a UV spectrophotometer. (A) Acetone:distilled water (1:1, v/v), λ_max_: 326 nm; (B) PBS, pH 7.4, containing 1% Tween 80, λ_max_: 307 nm.

After the devised UV–visible spectrophotometric technique was validated in PBS, pH 7.4, containing 1% Tween 80, linearity of *y* = 0.0617*x* + 0.0147 (*r*^2^ = 0.9991) was found in a concentration range of 2–12 μg·mL^–1^. RSD values of less than 2% for repeatability and moderate accuracy led to the determination that the procedure was exact. The method’s recovery and accuracy were deemed adequate due to an RSD value of less than 2%. Accuracy values of 100.008% ± 0.234, 100.062% ± 0.412, and 100.021% ± 0.510 for concentrations of 4, 6, and 8 μg·mL μg·mL^–1^, respectively, were determined for the UV–visible spectrophotometric method. Blank formulations (A-blank) were photometrically analyzed between 200 and 800 nm in the selectivity research, and they did not produce an absorbance peak at 307 nm. LOD and LOQ were obtained as 0.377 and 1.144 μg·mL^–1^, respectively, and proved its suitability by being lower than the lowest concentration studied in linearity. As a result, routine and simultaneous FA determination can be accomplished using the simple and affordable approach suggested in this study.^64^ Spectrum taken at 326 and 307 nm for FA are presented in Figure 2.

### Encapsulation Efficiency (EE, %)

The EE % of the A-FA coded formulation prepared in this study was obtained as 76.48% ± 3.12. The high and ideal EE % values obtained for the formulation coded A-FA can be explained as due to the low affinity of FA to the water phase and thus its lipophilic chemistry, which tends to migrate to the organic phase.^65^ High EE % provides the advantage of administering a lower amount of NPs to the patient for a given dose.^66,67^ According to the literature, it can be said that the 76.48% ± 3.12 value obtained for EE is ideal for oral administration.

### *In Vitro* Release

**Figure 3:**
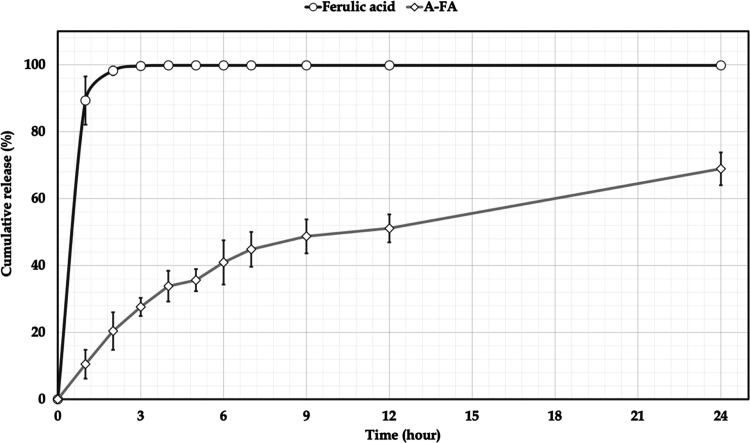
*In vitro* dissolution/release profile of FA and A-FA.

*In vitro* release profiles of pure FA and A-FA coded formulations are shown in Figure 3. In light of the gastrointestinal stability evaluation study and literature information carried out in this study, it was decided to perform the release test in a pH 7.4 PBS containing 1.0% Tween 80. In the release test, pure FA showed a release rate of 89.3% ± 7.9 at the end of the first hour and 98.2% ± 1.3 at the end of the second hour in the dissolution medium. At the end of the third hour, almost all of the FA was released in the dissolution medium, with a release rate of 99.6% ± 0.3. Considering the ferulic acid release rates from the A-FA arm nanoparticle formulation containing ferulic acid, values of 10.5 ± 4.3%, 20.4 ± 5.6%, 27.6 ± 2.7%, 33.8 ± 4.6%, 35.6 ± 3.3%, 40.9 ± 6.6%, 44.8 ± 5.2%, 48.7 ± 5.1%, 51.1 ± 4.2% and 68.9 ± 4.9% were obtained respectively, at the end of 1, 2, 3, 4, 5, 6, 7, 9, 12 and 24 h. When the literature is examined, it is quite clear that when the release of FA from the A-FA coded NP formulation is compared to pure FA, the A-FA coded NP formulation has a slower and 24-h extended-release.^68,69^

### *In Vitro* Release Kinetics

*R*^2^, *R*^2^_adjusted_, MSC, and AIC values obtained in the release kinetics study for the formulation coded A-FA are presented in Table 5. The release kinetics of FA from the A-FA coded NP formulation were found to be very similar to the Peppas–Sahlin and Weibull models. Stated differently, there was a strong correlation found between the Weibull model and the Peppas–Sahlin model. Consequently, this study’s findings demonstrate that Fickian (pure diffusion phenomena) and non-Fickian (relaxation of polymer chains between NPs) release processes, rather than a single mechanism, are primarily responsible for controlling the release of FA from NPs. When the literature is examined, similar results are found.^70^

**Table 5:** Release kinetics results for the A-FA

model and equation	*R*^2^	*R*_adjusted_^2^	MSC	AIC
**Zero order**	0.009	0.009	–0.191	80.119

**Zero order (*T***_**lag**_**)**	0.833	0.812	1.391	64.303

**Zero order (*F***_**0**_**)**	0.833	0.812	1.391	64.303

**First order**	0.735	0.735	1.127	66.937

**First order (*T***_**lag**_**)**	0.909	0.898	1.999	58.217

**First order (*F***_**max**_**)**	0.959	0.954	2.794	50.273

**First order (*T***_**lag**_**ve *F***_**max**_**)**	0.979	0.973	3.261	45.601

**Higuchi**	0.953	0.953	2.865	49.561

**Higuchi (*T***_**lag**_**)**	0.918	0.908	2.104	57.170

**Higuchi (*F***_**0**_**)**	0.956	0.951	2.728	50.930

**Korsmeyer-Peppas**	0.934	0.925	2.311	55.099

**Korsmeyer-Peppas (*T***_**lag**_**)**	0.936	0.918	2.155	56.657

**Korsmeyer-Peppas (*F***_**0**_**)**	0.782	0.719	0.922	68.993

**Hixson-Crowell**	0.584	0.584	0.677	71.440

**Hixson-Crowell (*T***_**lag**_**)**	0.887	0.873	1.780	60.413

**Hopfenberg**	0.678	0.638	0.733	70,881

**Hopfenberg (*T***_**lag**_**)**	0.887	0.855	1.580	62.413

**Baker-Lonsdale**	0.970	0.970	3.315	45.059

**Baker-Lonsdale (*T***_**lag**_**)**	0.965	0.960	2.945	48.763

**Peppas-Sahlin 1**	0.963	0.953	2.709	51.118

**Peppas-Sahlin 1 (*T***_**lag**_**)**	0.986	0.979	3.471	43.503

**Peppas-Sahlin 2**	0.970	0.967	3.119	47.020

**Peppas-Sahlin 2 (*T***_**lag**_**)**	0.989	0.986	3.936	38.849

**Weibull 1**	0.987	0.983	3.736	40.851

**Weibull 2**	0.973	0.970	3.213	46.081

**Weibull 3**	0.984	0.980	3.556	42.655

**Weibull 4**	0.987	0.980	3.518	43.026


### Solid-State Characterization

#### Thermal Analysis

**Figure 4:**
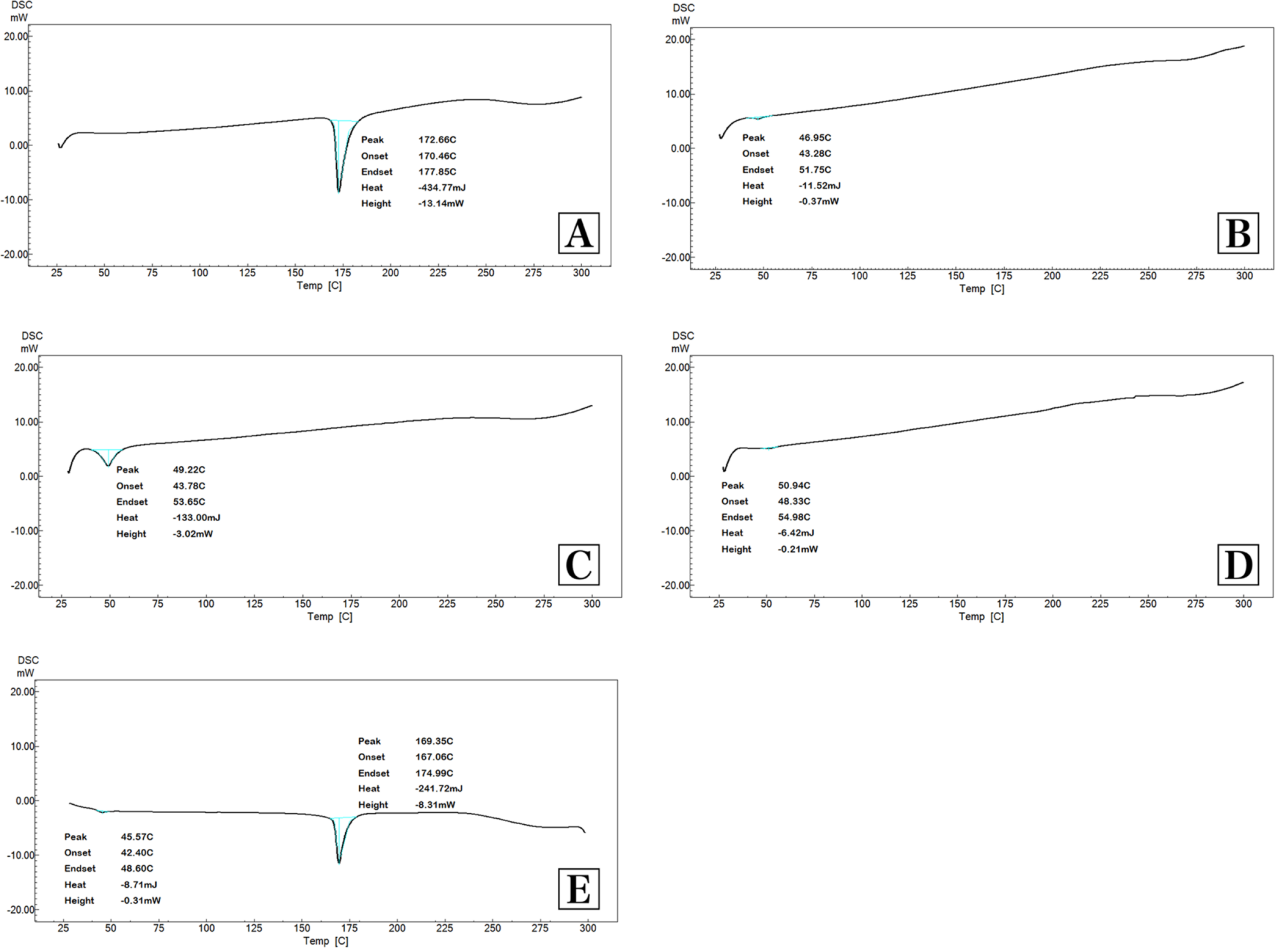
DSC analysis results. (A) Ferulic acid, (B) PLGA, (C) A-Blank, (D) A-FA, and (E) physical mixture.

DSC curves of FA, PLGA, A-FA, A-Blank, and physical mixture are given in Figure 4. Evidence that the encapsulation process was successful can be obtained from DSC thermograms showing the thermal transition profile for the formulations.^71^ Pure FA exhibited a melting peak at 172.66 °C. When the literature is examined, this characteristic endothermic melting peak is suitable for FA.^72^ In the formulations coded A-Blank and A-FA, the endothermic peak/melting point was observed at 49.22 and 50.94 °C, respectively; these peaks are an indicator of the glass transition temperature of PLGA.^42^ In the physical mixture, both the characteristic melting peaks of FA and the peaks belonging to the glass transition temperature of PLGA were observed. The disappearance of the FA melting peak in the A-FA coded NP formulation’s thermogram indicated that FA was diffused molecularly throughout the polymeric framework and contained in its amorphous state.^73,74^ Furthermore, the thermogram demonstrated that FA and the polymers did not interact. These data obtained are important because the presence of the drug in molecular dispersion form helps with its extended-release feature.^75^

#### Fourier-Transform Infrared Spectroscopy (FT-IR) Analysis

**Figure 5:**
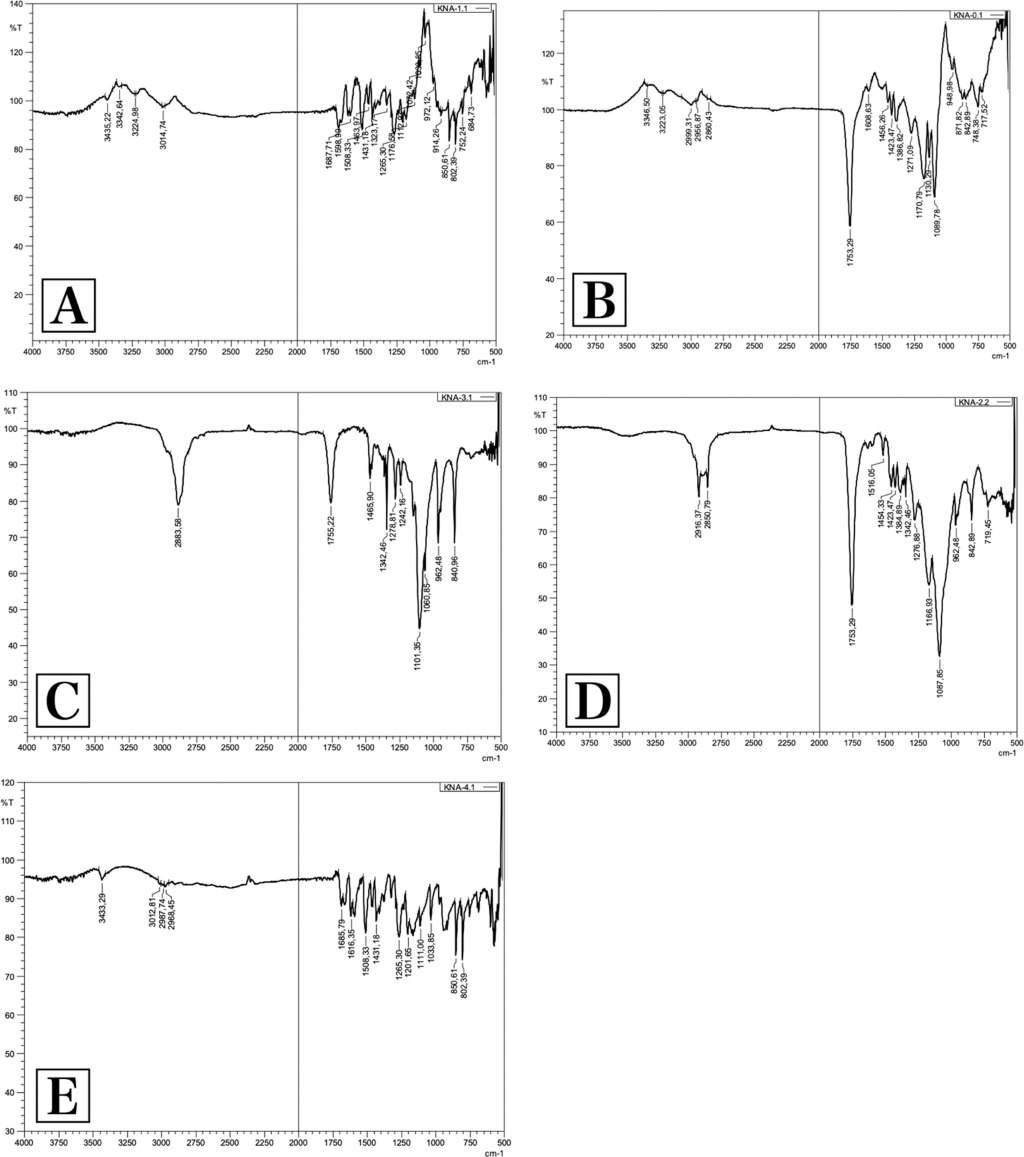
FT-IR analysis results. (A) Ferulic acid, (B) PLGA, (C) A-Blank, (D) A-FA, and (E) physical mixture.

FT-IR analysis results are given in Figure 5. Pure FA showed a peak at 3435.22 cm^–1^, which is typical for – OH stretching vibrations. The absorption bands around 3014.74 cm^–1^ correspond to the presence of alkane groups. The band at 1687.71 cm^–1^ was observed for the C=O carbonyl group and the band at 1265.30 cm^–1^ was observed for the C-O group. The signals at 1598.99 and 1508.33 cm^–1^ are related to the vibration of the aromatic ring, while the peak around 1200.00 cm–^1^ is typical for C-OH stretching and finally the band at 1033.85 cm^–1^ is typical for methoxide O-CH_3_ stretching. In line with these data, the FT-IR spectrum of pure FA was found to be compatible with the literature.^35,71^

When the spectrum of PLGA was examined, characteristic absorption bands of PLGA were noticed. The bands at 3346.50 cm^–1^ are typical for hydroxyl groups, and the bands at 2999.31 cm^–1^ correspond to the vibration for C-H alkane groups. Characteristic stress peaks for the C=O carbonyl group were observed at 1753.29 cm^–1^. The bands between 1300 and 1400 cm^–1^ are characteristic of the bending vibration of C-H alkane groups, and the bands in the region between 1271.09 and 1089.78 cm^–1^ are characteristic of C-O vibration. In line with these data, the FT-IR spectrum of PLGA was found to be compatible with the literature.^72^

The FT-IR spectrum of the A-Blank coded formulation without an active ingredient was obtained like PLGA. In the physical mixture, characteristic peaks of both FA and PLGA were observed. As supported by the DSC results, a significant decrease in FA peaks was observed in the spectra of the A-FA coded FA-loaded NP formulation, which shows the molecular distribution of FA in polymeric matrices, and this confirmed the encapsulation of FA in the polymeric structure.^76,77^

#### Nuclear Magnetic Resonance (^1^H NMR) Analysis

**Figure 6:**
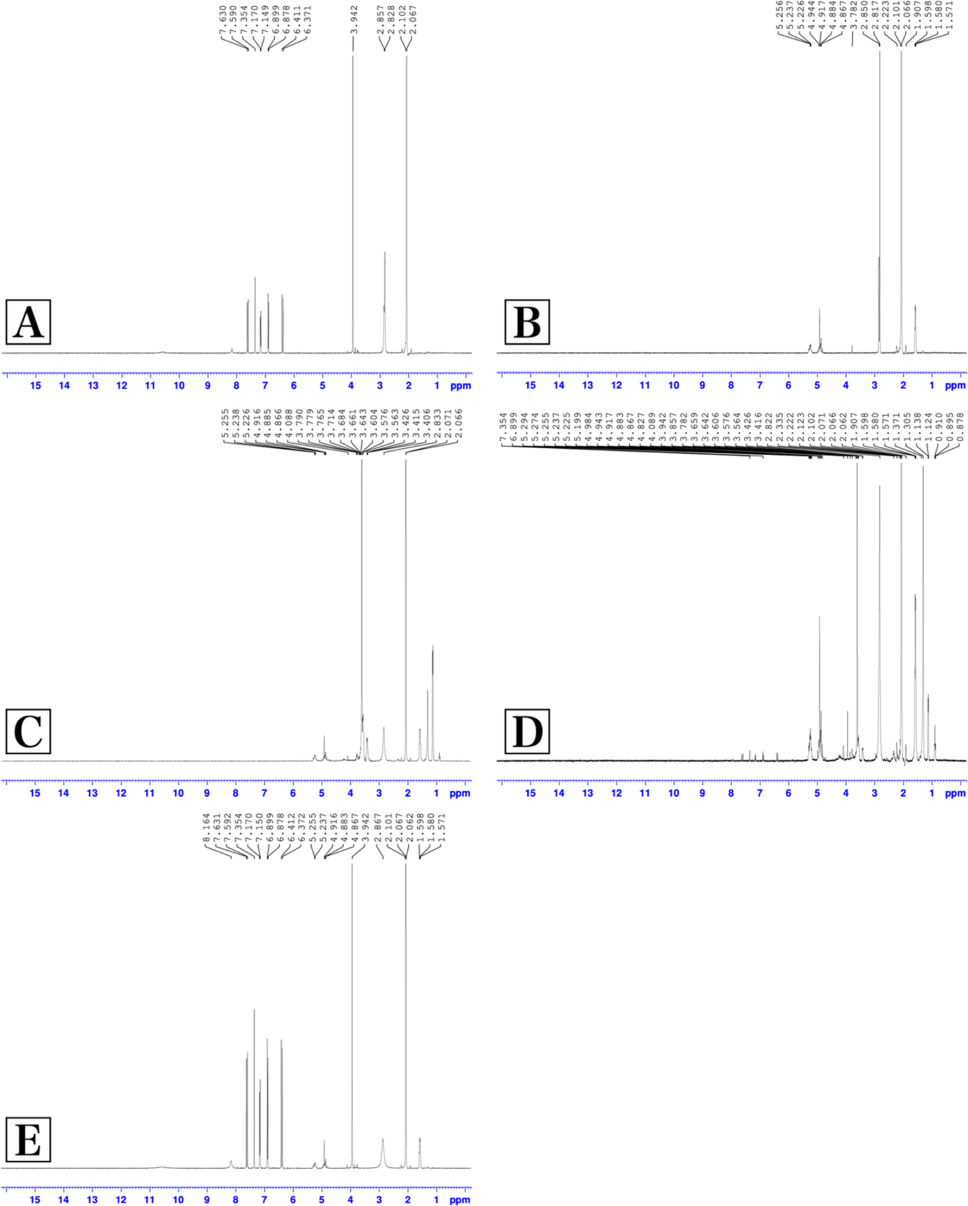
^1^H NMR analysis results. (A) Ferulic acid, (B) PLGA, (C) A-Blank, (D) A-FA, and (E) physical mixture.

^1^H NMR analysis results are given in Figure 6. In a study conducted with FA, it was emphasized that specific peaks of FA were observed at 7.49, 7.20, 7.08, 6.69, 6.37, and 3.82 ppm. When the ^1^H NMR spectrum of FA was examined, it was found to be compatible with the literature.^78^ In this study, the spectrum obtained from the ^1^H NMR analysis of the blank formulation (A-Blank) prepared without active ingredient was similar to the spectrum of pure polymer (PLGA), and no peak was found at the ppm points and ranges where the specific peaks of FA come.^43^ In the formulation coded A-FA prepared with the active ingredient, characteristic peaks of FA were observed, but the density decreased. The molecular distribution of FA and the fact that it peaks at characteristic ppm values with low intensity in proportion to the concentration suggest that FA is molecularly distributed within the polymeric structure. This situation was also interpreted as FA being successfully loaded into NPs.^58^

### Biological Activity

ABTS radical cation decolorization method results are presented in Table 6. According to the activity results, A-Blank does not show activity compared to BHT and ascorbic acid, while FA-loaded formulation coded A-FA and FA showed significant ABTS cation decolorizing antioxidant activity. The activity order was obtained as A-FA > FA > ascorbic acid > BHT.

**Table 6:** *In Vitro* ABTS Radical Decolorization Activity of Samples

sample	IC_50_ (mg·mL^–1^)
ferulic acid	0.2454
A-FA	0.2146
A-blank	nd
Vit C (ascorbic acid)	0.2545
BHT	0.4325

nd: no activity detected.

Reducing power method results are presented in Table 7. According to the activity results, when compared to BHT and ascorbic acid, the A-FA coded formulation showed the highest reducing power, while FA showed the lowest activity. The formulation coded A-Blank did not show any effect. The activity order was obtained as ascorbic acid > A-FA > BHT > FA.

**Table 7:** *In Vitro* Reducing Powers of Samples

sample	IC_50_ (mg·mL^–1^)
ferulic acid	0.1721
A-FA	0.097
A-Blank	nd
Vit C (ascorbic acid)	0.085
BHT	0.1564

nd: no activity detected.

The results of the DPPH free radical scavenging method are presented in Table 8. According to the activity results, A-FA showed the highest DPPH free radical scavenging antioxidant activity compared to BHT, while FA showed the lowest activity. The formulation coded A-Blank did not show any effect. The activity ranking was obtained as A-FA > FA > BHT. As a result, according to the antioxidant activity results, A-FA showed strong DPPH free radical scavenging, ABTS cation decolorizing, and reducing antioxidant activity.

**Table 8:** *In Vitro* Antioxidant-DPPH Radical Scavenging Activity of Samples

sample	IC_50_ (mg·mL^–1^)
ferulic acid	0.00044
A-FA	0.00040
A-Blank	nd
BHT	0.0011

nd: no activity detected.

AChE inhibitor activity results are given in Table 9. According to the activity results, when compared to the acetylcholinesterase inhibitor donepezil hydrochloride, the A-FA coded formulation showed the highest anti-AChE activity with an IC_50_ of 121.65 μg·mL^–1^, while A-Blank showed the lowest activity. The activity order was obtained as A-FA > donepezil hydrochloride > FA > A-Blank. BuChE inhibitor activity results are given in Table 10. According to the activity results, compared to donepezil hydrochloride, A-FA showed the highest anti-BChE activity with an IC_50_ of 419.46 μg·mL^–1^, while FA showed the lowest activity. A-Blank had no effect. The activity order was obtained as donepezil hydrochloride > A-FA > FA. As a result, according to the anti-Alzheimer activity results, A-FA showed strong anti-AChE activity compared to Donepezil HCl, which is an acetylcholinesterase inhibitor (IC_50_ = 121.65 μg·mL^–1^).

**Table 9:** AChE Inhibitor Activity Results

sample	IC_50_ (μg·mL^–1^)
ferulic acid	407.497
A-FA	121.65
A-Blank	426.80
donepezil HCl	206.186

**Table 10:** BuChE Inhibitor Activity Results

sample	IC_50_ (μg·mL^–1^)
ferulic acid	519.75
A-FA	419.46
A-Blank	nd
donepezil HCl	220.750

nd: no activity detected.

It has been established that FA has an antioxidant impact against a range of acute and chronic illnesses, including intestinal ischemia, cancer, cardiovascular and skin disorders, diabetes, oxidative cellular stress in human dermal fibroblasts, and cochlear oxidative damage from repeated noise exposure. Furthermore, FA’s ability to scavenge free radicals has been evaluated against a variety of neurodegenerative diseases, including AD.^79^

Various findings in the medical field report the onset of several diseases associated with free radical production. Oxidative stress is known to cause reactive molecules called free radicals to accumulate in the body and cause oxidative damage. This condition is associated with various factors such as unhealthy lifestyles, chemical exposure, pollution, smoking, drug use, some diseases, and stress. Although oxygen is essential for many biological processes, oxidative events can exacerbate intracellular damage. Oxygen is the main source of energy, and free radicals are formed because of adenosine triphosphate (ATP) produced by the mitochondria. The principal byproducts of cellular redox reactions are reactive nitrogen species (RNS) and reactive oxygen species (ROS). Depending on their delicate balance within cells, these reactive species can be beneficial or toxic compounds. At low or moderate levels, reactive species exert beneficial effects on cellular redox signaling and immune function, whereas at high concentrations, they can produce oxidative stress and cause a harmful process that can damage cellular function and structure. Antioxidants are substances that can scavenge free radicals and maintain cellular redox balance, helping to reduce the incidence of damage that causes oxidative stress.^80,81^

It is well-recognized that exogenous antioxidant consumption improves human health and effectively lowers the prevalence of diseases caused by free radicals, such as neurological illnesses.^79^ Oxidative stress has been found to contribute to Alzheimer’s neuropathology.^82^ For this reason, both antioxidant activity studies and anti-Alzheimer activity studies were conducted with the A-FA coded NP formulation prepared within the scope of this study. According to the antioxidant activity results, FA-loaded A-FA coded NP formulation showed strong DPPH free radical scavenging, ABTS cation decolorizing, and reducing antioxidant activity.

Inhibition of AChE and BuChE is the target in the effective treatment of AD by reducing β-amyloid accumulation in the brain and increasing acetylcholine utilization.^83^ AChE inhibitory effect and BuChE inhibitory effect studies were carried out to investigate the potential of the A-FA coded optimum formulation prepared within the scope of this thesis to be used in AD. In the AChE inhibitor effect study, the IC_50_ value of the A-FA coded formulation loaded with FA was obtained as 121.65 μg·mL^–1^, and in the BuChE inhibitor effect study, the IC_50_ value was found to be 419.46 μg·mL^–1^. As a result, according to the anti-Alzheimer activity results, A-FA showed strong anti-AChE activity compared to Donepezil HCl, which is an acetylcholinesterase inhibitor. The damage caused by free radicals caused by oxidative stress to neurons and metal accumulation in the brain is directly related to the pathogenesis of Alzheimer’s. It is important in the treatment that a therapeutic agent used against Alzheimer’s has both AChE inhibitor and antioxidant properties.^83^

## Conclusions

It is known that substances with antioxidant properties have a positive effect on the treatment of many diseases, especially Alzheimer’s disease. Even though there have been numerous studies on ferulic acid in recent years, there have been relatively few investigations into the antioxidant and anti-Alzheimer effects of the resulting formulations, polymeric systems, and drug delivery systems. For this reason, in this study, ferulic acid-loaded PLGA-based nanoparticles were prepared by the ’nanoprecipitation’ method, and the effects of Poloxamer 188 concentration in the aqueous phase and Span 60 concentration in the organic phase on the nanoparticle properties were examined. An increase in particle size was detected with increasing Poloxamer 188 and Span 60 concentration. To determine the point and not to use too much excipient, the A-Blank coded formulation was chosen as optimum and a ferulic acid-loaded version was prepared. The particle size of the ferulic acid-loaded A-FA coded nanoparticle formulation was obtained as 174.70 nm ± 0.89, and it was proven that the formulation was monodisperse with a PDI value of 0.113 ± 0.006. The ζ potential value was obtained as – 22.00 mV ± 0.56, and it was concluded that it could be stable for a long time. The effect of trehalose on the formulation coded A-FA was evaluated, and storage conditions were determined. In the 24-h stability test performed in gastrointestinal fluids, it was concluded that the formulation coded A-FA degraded faster in acidic environments. Due to the low affinity of ferulic acid to the water phase and thus its tendency to migrate to the organic phase, high encapsulation efficiency was achieved, and the encapsulation efficiency was obtained as 76.48 ± 3.12%. When the release of ferulic acid from the A-FA coded nanoparticle formulation was compared with pure ferulic acid, it was determined that the A-FA coded nanoparticle formulation had a slower and extended release of 24 h. In line with the results obtained with DDSolver, a high correlation was observed between the Peppas–Sahlin model and the Weibull model, and it was concluded that the release kinetics were not formed by a single specific mechanism but by a combined Fickian and non-Fickian mechanism. Encapsulation was proven by DSC, FT-IR, and ^1^H-NMR analyses. After the formulation coded A-FA was characterized in terms of pharmaceutical technology, biological activity studies were carried out. According to the antioxidant activity results, ferulic acid-loaded A-FA coded nanoparticle formulation showed strong DPPH free radical scavenging, ABTS cation decolorizing, and reducing antioxidant activity. According to the anti-Alzheimer activity results, A-FA showed strong anti-AChE activity compared to Donepezil HCl, which is an acetylcholinesterase inhibitor. Since it is important for a therapeutic agent used against Alzheimer’s to have both AChE inhibitor and antioxidant properties, it has been concluded that the formulation prepared in this study is promising in the treatment of both oxidative stress-related diseases and Alzheimer’s. In the later stages of the study, it is planned to carry out planned long-term stability tests in accordance with ICH guidelines, conduct *in vitro* and *in vivo* blood–brain barrier passage studies, conduct angiogenesis studies with the help of chorioallantoic membrane (CAM) tests, and characterize the A-FA coded formulation with different *in vivo* Alzheimer’s models.
